# Musculoskeletal spine modeling in large patient cohorts: how morphological individualization affects lumbar load estimation

**DOI:** 10.3389/fbioe.2024.1363081

**Published:** 2024-06-12

**Authors:** Tanja Lerchl, Kati Nispel, Jannis Bodden, Anjany Sekuboyina, Malek El Husseini, Christian Fritzsche, Veit Senner, Jan S. Kirschke

**Affiliations:** ^1^ Associate Professorship of Sports Equipment and Sports Materials, School of Engineering and Design, Technical University of Munich, Garching, Germany; ^2^ Department of Diagnostic and Interventional Neuroradiology, School of Medicine, Klinikum Rechts der Isar, Technical University of Munich, Munich, Germany; ^3^ Department of Quantitative Biomedicine, University of Zurich, Zurich, Switzerland

**Keywords:** spinal biomechanics, multi body dynamics, subject-specific modeling, individualization, automated model generation, spinal loading

## Abstract

**Introduction:** Achieving an adequate level of detail is a crucial part of any modeling process. Thus, oversimplification of complex systems can lead to overestimation, underestimation, and general bias of effects, while elaborate models run the risk of losing validity due to the uncontrolled interaction of multiple influencing factors and error propagation.

**Methods:** We used a validated pipeline for the automated generation of multi-body models of the trunk to create 279 models based on CT data from 93 patients to investigate how different degrees of individualization affect the observed effects of different morphological characteristics on lumbar loads. Specifically, individual parameters related to spinal morphology (thoracic kyphosis (TK), lumbar lordosis (LL), and torso height (TH)), as well as torso weight (TW) and distribution, were fully or partly considered in the respective models according to their degree of individualization, and the effect strengths of these parameters on spinal loading were compared between semi- and highly individualized models. T-distributed stochastic neighbor embedding (T-SNE) analysis was performed for overarching pattern recognition and multiple regression analyses to evaluate changes in occurring effects and significance.

**Results:** We were able to identify significant effects (*p* < 0.05) of various morphological parameters on lumbar loads in models with different degrees of individualization. Torso weight and lumbar lordosis showed the strongest effects on compression (*β* ≈ 0.9) and anterior–posterior shear forces (*β* ≈ 0.7), respectively. We could further show that the effect strength of individual parameters tended to decrease if more individual characteristics were included in the models.

**Discussion:** The induced variability due to model individualization could only partly be explained by simple morphological parameters. Our study shows that model simplification can lead to an emphasis on individual effects, which needs to be critically assessed with regard to *in vivo* complexity. At the same time, we demonstrated that individualized models representing a population-based cohort are still able to identify relevant influences on spinal loading while considering a variety of influencing factors and their interactions.

## 1 Introduction

Chronic back pain is a multi-factorial problem (Murtezani et al., 2011; Rabey et al., 2019). Apart from psychological and social causes (Tagliaferri et al., 2020), spinal degeneration is often associated with back pain, but it also comes with a variety of possible sources itself (Kalichman et al., 2009). Thus, age- or disease-related changes in passive structures can lead to pain and disability, as well as individual anthropometric conditions, such as body weight or spinal alignment and deformities (Kalichman et al., 2017). *In vivo* investigations on spinal loading are rare and usually consider single individuals and spinal levels (Wilke et al., 2001; Takahashi et al., 2006; Rohlmann et al., 2008), providing the necessary basis for model validation but being unsuitable for comparative studies on the potential influences of inter-individual characteristics on spinal loads.

For systematic analyses of spine biomechanics, numerical modeling and simulation have been widely established in the past few years (de Zee et al., 2007; Christophy et al., 2012; Bruno et al., 2015; Ignasiak et al., 2016). Although finite element simulation is primarily suitable for the examination of deformation states and internal stresses in single flexible bodies (Périé et al., 2002; Little and Adam, 2015; Ghezelbash et al., 2016a; El Ouaaid et al., 2016; Naserkhaki et al., 2016; Vergari et al., 2016; Akhavanfar et al., 2018; Eskandari et al., 2019), multi-body modeling allows the consideration of the biomechanics of the spine from a more comprehensive perspective and can take multiple aspects of mechanical loading into account (Lerchl et al., 2023). However, the vast majority of published studies use generic models to focus on the effects of factors such as sagittal alignment (Bruno et al., 2012; Bruno et al., 2017; Galbusera et al., 2014; Bassani et al., 2019; Müller et al., 2021) or body weight (Akhavanfar et al., 2018). Although those studies are inevitable to examine isolated effects of the parameters of interest, they fail to capture the complexity of clinical practice. Each patient comes with a unique combination of influencing factors that interact with each other and lead to individual loading scenarios. To address this complexity, a recent trend toward individualized models has emerged in the relevant literature (Burkhart et al., 2020; Overbergh et al., 2020; Fasser et al., 2021; Lerchl et al., 2022; Banks et al., 2023). Individualized modeling is usually time-consuming, and therefore, respective models are often only available in small sample sizes. However, in order to obtain meaningful and statistically significant results, the analysis of large and diverse patient cohorts is essential. Due to diagnostic and clinical practice as well as large population-based cohort studies (e.g., the German National Cohort and the UK Biobank), such datasets are available for scientific interest, and during the past decade, developments in data analytics—especially in the field of artificial intelligence—have been providing promising tools to make these datasets accessible for further analysis (Sekuboyina et al., 2020).

For all the potential that individualized models hold, they also pose special challenges. The balancing act between sufficient model complexity and necessary simplifications is an integral part of any modeling process, enabling us to draw distinct conclusions from the obtained results. Taking into account multiple individual characteristics inevitably increases model complexity, carries the risk of generating noise, increases result variance, and therefore makes it difficult to draw clear conclusions. On the other hand, oversimplified models can also lead to biased results, such as the overestimation of individual effects due to the neglect of other parameters and their interrelations. The question of the right level of detail is, therefore, crucial in biomechanical modeling. To the best of our knowledge, there are no musculoskeletal modeling studies published that examine the effects of multiple parameters on spinal loading based on a large patient cohort and critically analyze how different degrees of individualization influence simulation results using a population-based cohort.

We used a pipeline for the automated generation of individualized multi-body models of the trunk (Lerchl et al., 2022) to investigate the influence of different degrees of model individualization on the observed effects of morphological factors on spinal loading. We analyzed how the effects of individual parameters on spinal loading change with the increasing degree of individualization of the underlying models. Highly individualized models included a patient-specific spine as well as torso weight (TW) and its distribution, which was combined with a generic pelvis, sacrum, ribcage, head–neck, and simplified arms. We carried out analyses based on a large patient cohort representing a diverse population in terms of spinal morphology and alignment as well as torso weight and its distribution (*n* = 93, M = 55, F = 38, and age = 70 ± 7.6) (Table 1). Parameters of interest were thoracic kyphosis (TK), lumbar lordosis (LL), torso height (TH)TW, and left of mass of the torso in anterior–posterior and superior–inferior directions (CoM AP and CoM SI). According to the degree of individualization, we combined individualized and uniform representations of those parameters for different model configurations.

**TABLE 1:** Summary of average dataset characteristics, namely, the sample size (n), age, TK, LL, TH, TH, CoM AP, and CoM SI in reference to the sacrum.

	n	Age	Av. TK [°]	Av. LL [°]	Av. TH [m]	Av. TW [kg]	Av. CoM AP [m]	Av. CoM SI [m]
Full	93	70.0 ± 7.6	42.2 ± 11.5	37.4 ± 12.0	0.43 ± 0.03	25.1 ± 5.9	0.03 ± 0.01	0.21 ± 0.01
Males	55	70.9 ± 7.1	42.0 ± 11.6	36.3 ± 11.7	0.45 ± 0.02	27.0 ± 5.1	0.03 ± 0.01	0.21 ± 0.01
Females	38	68.6 ± 8.1	42.6 ± 11.5	38.9 ± 12.3	0.41 ± 0.02	22.4 ± 5.9	0.02 ± 0.01	0.20 ± 0.01

## 2 Methods

### 2.1 Musculoskeletal modeling and simulation

We used our pipeline for the automated generation of individualized musculoskeletal models of the trunk, including upper extremities and head–neck, to segment vertebral geometries, as well as torso weight and distribution, from computed tomography (CT) scans of 93 patients. Therefore, we labeled and segmented vertebrae using an automated deep learning-based process (Sekuboyina et al., 2020) and subsequently derived individual body weight and distribution, spinal alignment, and points of interest for muscle and ligament attachments (Lerchl et al., 2022). A medical professional clinically assessed TK and LL based on the imaging data of each patient.

TH was measured from the upper endplate of T1 to the lower endplate of L5. TW and CoM were calculated from the soft tissue segmentation derived from the imaging data. Torso weight was subdivided into segments for each vertebral level. For each segment, the algorithm calculated its left of mass and total weight, corresponding to its individual tissue distribution. We assume an average density of 0.25 g/cm^3^ for the lungs, 0.96 g/cm^3^ for fat, and 1.06 g/cm^3^ for the remaining soft tissues (Pearsall et al., 1996; Akhavanfar et al., 2018). Subsequently, we calculated the CoM across all levels, considering its anterior (AP) and superior (SI) directions in reference to L5 in our analysis. An overview of the sample characteristics is summarized in Table 1.

**FIGURE 1:**
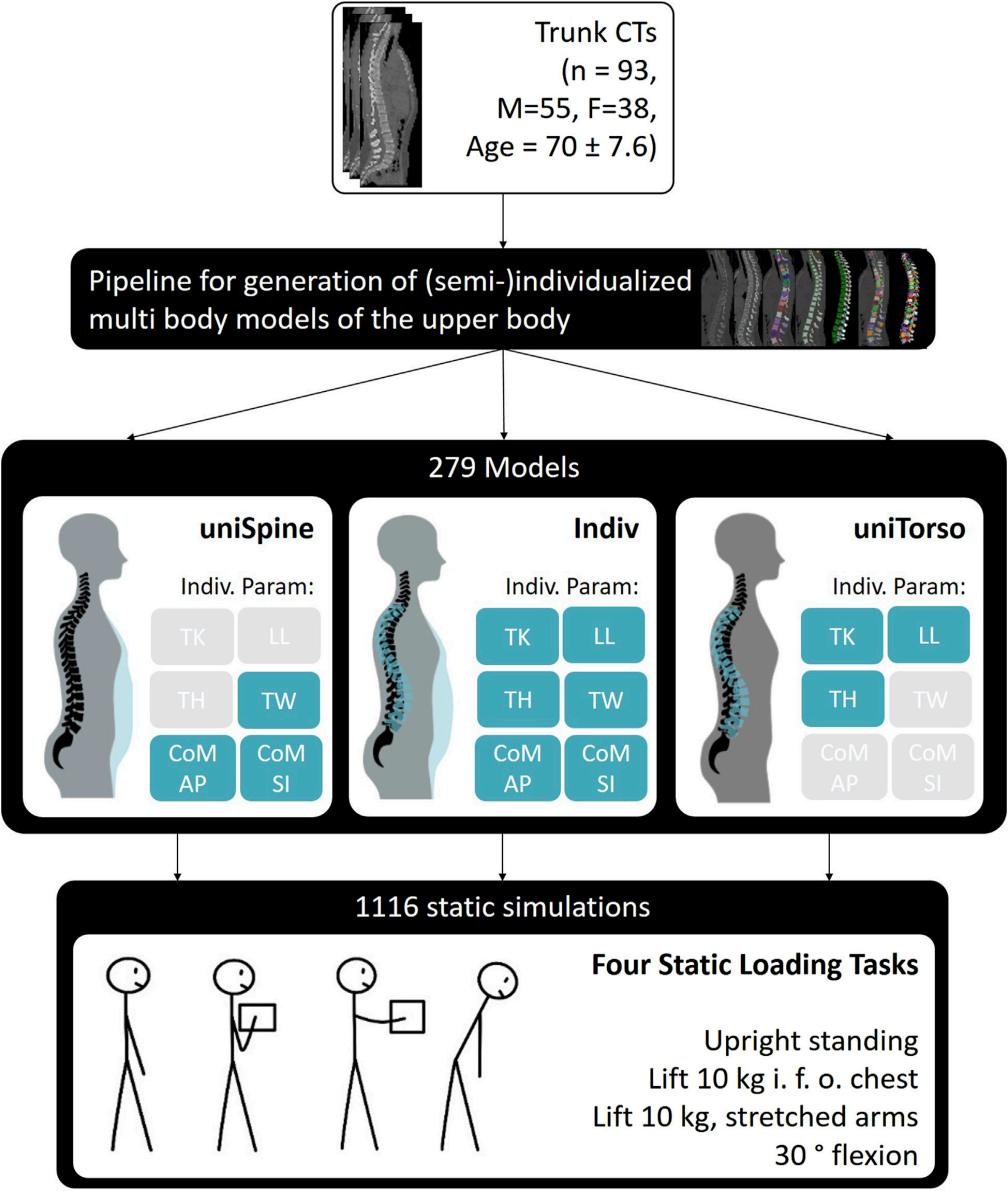
Model generation based on 93 patients with different degrees of individualization. For each of the overall 279 models, 4 static loading tasks were simulated.

For each patient, we generated three models using multi-body simulation software SIMPACK 2023x (Dassault Systèmes, France): one model with individualized spine and torso (Indiv), one with uniform spine and individualized torso (uniSpine), and one with uniform torso (uniTorso) and individualized spine. The uniform spine was derived from patient data, representing the average healthy spine of a 67-year-old male (TK = 29°, LL = 44°, and TH = 0.45 m). The uniform torso weight was customized to a TW of 23.3 kg, with fixed distribution and moment arms for each level along the thoracolumbar spine. More precisely, highly individualized models (Indiv) describe models with patient-specific spine anatomy and torso weight and distribution. Semi-individualized models, respectively, only include individualized spinal anatomy (uniTorso) and individualized torso weight and distribution (uniSpine) (Figure 1).

**FIGURE 2:**
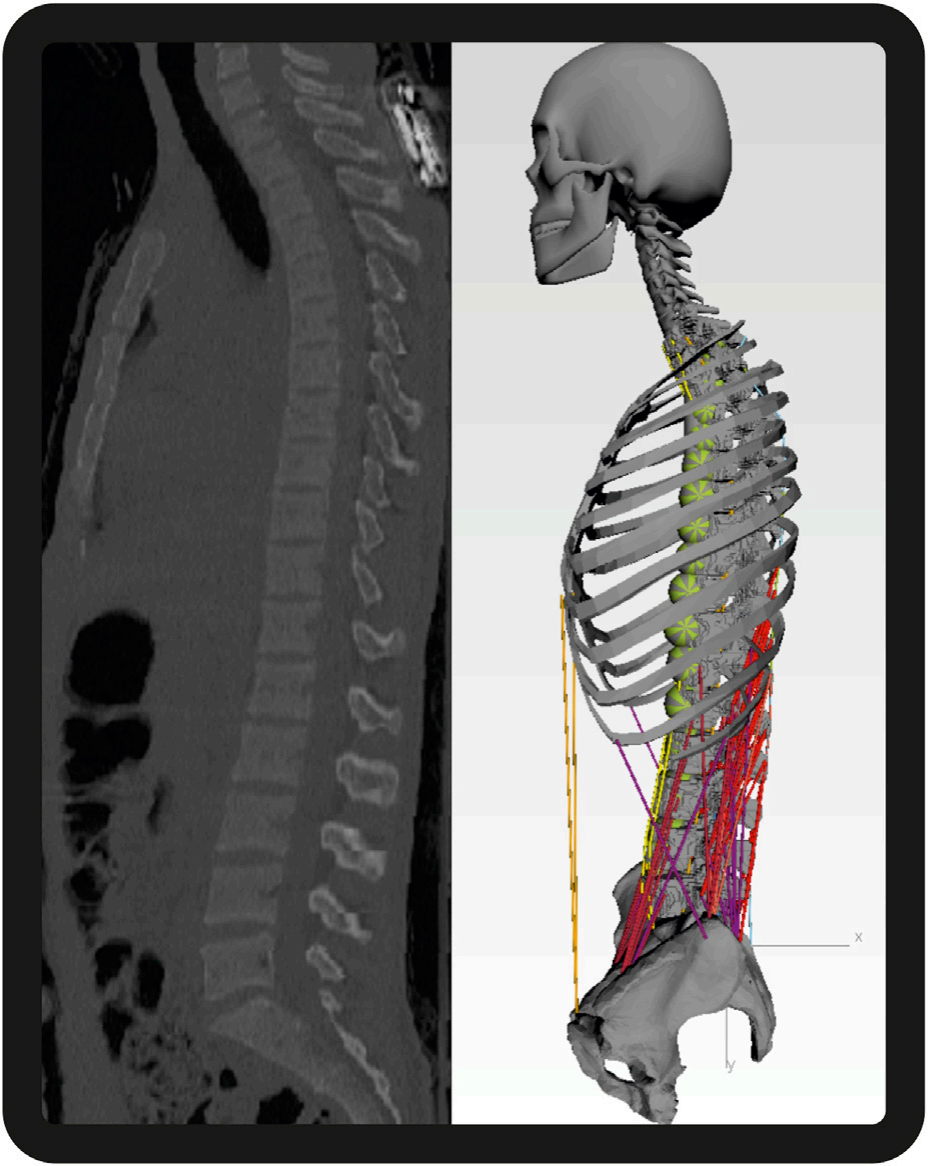
Sagittal CT images of one subject with the respective MBS model. Segment masses for soft tissues are visualized by the green spheres. For the sake of clarity, the dummy bodies for the arms consisting of two scaled cylinders centrally attached to T3 are not shown here [adapted from Lerchl et al. (2022)].

All models further included generic bodies for the head–neck, ribcage, sacrum, pelvis, and simplified arms (Figure 2). Intervertebral discs and paraspinal ligaments are modeled as non-linear elastic elements. Intervertebral joints L1–L5 are modeled as spherical joints, and the thoracic spine and ribcage are simplified as one rigid body. We incorporated detailed generic muscle architecture for the lumbar spine, including the rectus abdominis (RA), internal oblique (IO), external oblique (EO), psoas major (PM), quadratus lumborum (QL), multifidus (MF), longissimus thoracis pars lumborum (LTL), iliocostalis lumborum (IL), and interspinales lumborum (IS), based on data from the literature (Christophy et al., 2012; Bayoglu et al., 2017).

We simulated four static loading tasks for each model, leading to a total of 1,116 simulations. The investigated load cases were three variations: upright standing in a neutral position, lifting 10 kg in front of the chest with a distance of 25 cm from T3 (10 kg, 25 cm), and lifting 10 kg with stretched arms with a distance of 55 cm (10 kg, 55 cm) and 30° flexion. Respective joint angles were assumed to be 40% sacral rotation and 60% lumbar flexion (Liu et al., 2019a), while lumbar flexion was distributed as 25.5% for L1/L2, 23.1% for L2/L3, 20.4% for L3/L4, 18.5% for L4/L5, and 12.5% for L5/S1 (Wong et al., 2006; Christophy et al., 2012). Muscle force estimation was carried out using combined inverse dynamics and static optimization, minimizing the sum of cubed muscle stress (Crowninshield and Brand, 1981). We defined inequality constraints to account for occurring moments in the intervertebral joints during each load case and bound constraints to set maximal muscle stress to 1 MPa (Bruno et al., 2015; Beaucage-Gauvreau et al., 2019; Favier et al., 2021). To account for changes in posture due to the supine position during CTs, a previous optimization was carried out to find the optimal neutral standing position by optimizing lumbosacral sagittal angles (Lerchl et al., 2022). Model validation was carried out based on *in vivo* studies (Wilke et al., 2001; Takahashi et al., 2006; Rohlmann et al., 2008) using two individualized models, showing a good overall correlation with measured spinal loads (r = 0.98) and muscle activity (r = 0.95). Model generation and validation are described in detail by Lerchl et al. (2022). Lumbar loads were evaluated based on compression and anterior–posterior shear forces, which were defined locally in reference to the respective functional spine unit (FSU). Thus, the compression force was assumed to be normal to the upper-end plate of the lower vertebra of the FSU, while the anterior–posterior shear force is defined in the midplane of the vertebra orthogonal to the compression force, pointing posteriorly.

### 2.2 Statistical analysis

First, we qualitatively examined our simulation results for potential overarching patterns across all lumbar levels. Therefore, we MinMax scaled absolute compression and shear forces under consideration of respective signs and applied t-distributed stochastic neighbor embedding (T-SNE) (Van der Maaten and Hinton, 2008), a statistical method that maps high-dimensional data to a virtual two- or three-dimensional space while preserving local similarities. Therefore, higher-dimensional data are converted into a visualizable space while concisely containing the underlying information. In other words, similar data points are clustered closely, while those that differ strongly are displayed with a matching distance. It is used for non-linear dimension reduction, pattern recognition, and visualization of high-dimensional data. In our case, this enabled us to visually analyze possible trends overarching all lumbar levels. We used the Python package scikit-learn for statistical analysis and applied T-SNE first to the complete dataset for compression and anterior–posterior shear forces individually, as well as for combined loading, including both components simultaneously. Using color mapping, we subsequently analyzed potential influences of considered individual factors (TK, LL, TH, TW, CoM AP, and CoM SI) across all lumbar levels in individualized models.

For quantitative load case- and level-specific analysis of possible influences on lumbar loading, we used multiple regression based on the least squares method. Independent variables were TK, LL, TH, TW, CoM AP, and CoM SI, while compression and anterior–posterior shear forces were defined as dependent variables. To ensure comparability despite different underlying scales, we lefted and standardized the data. We compared the regression coefficients *β* of the respective parameters of interest as a measure of the observed effect strength. The significance of the results was evaluated based on the determined *p*-value. Significance levels were set to 0.05 (*), 0.01 (**), and 0.001 (***). According to the individualized parameters in the respective models, we applied multiple regression with three independent variables related to spinal alignment (TK, LL, and TH) for uniTorso models and three independent variables related to torso weight and distribution (TW, CoM AP, and CoM SI) for uniSpine models. For highly individualized models (Indiv models), we performed multiple regression with all six independent variables. We investigated changes in the observed effect strength (*β*) and significance (*p*-value) of each parameter for different degrees of model individualization. To investigate how the inclusion of additional individual parameters affects the generated results, we compared the results from highly individualized models (Indiv) with those from only partly individualized models, namely, those with a uniform spine and individualized torso weight and distribution (uniSpine) or uniform torso and individualized spine (uniTorso). Furthermore, the coefficient of determination (*R* ^2^) was used to evaluate whether the included parameters were able to explain the observed variability.

## 3 Results

### 3.1 T-SNE analysis

**FIGURE 3:**
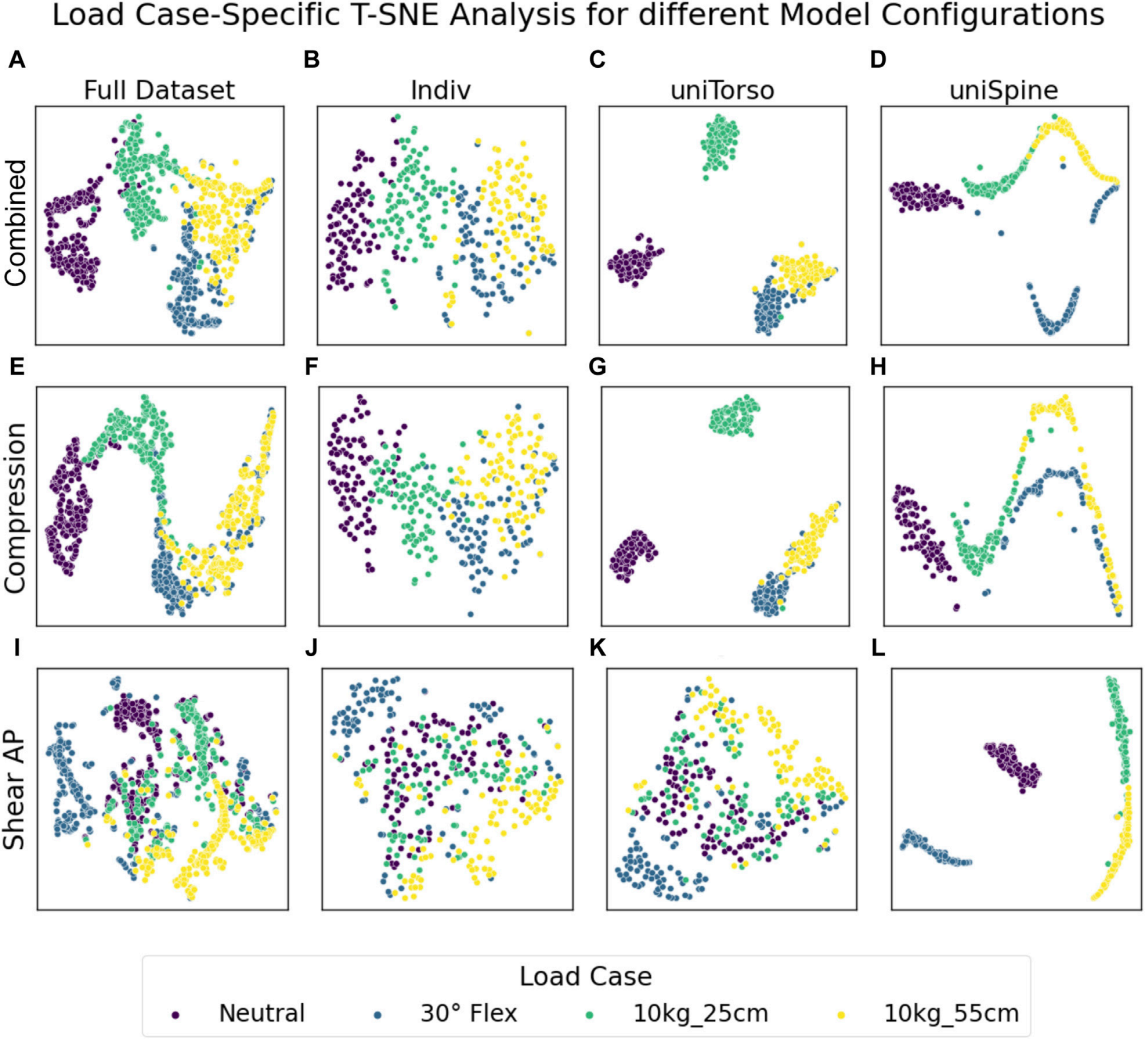
Load case-specific T-SNE analysis for the full dataset **(A, E, I)** and each model configuration isolated **(B–D, F–H, J–L)**. Semi-individualized models (uniSpine and uniTorso) showed concise clustering, especially in combined loading and compression **(C, D, G, H)**, while the inclusion of more individual parameters (Indiv) showed that results due to specific load cases were more similar to each other while still preserving clear clusters **(B, F)**. Note that only individualization of the spine **(K)** resulted in comparable scattering in anterior–posterior shear forces as individualization of both the torso and spine **(J)**. *X* and *y* axes represent a virtual space, which serves for projection and is not directly interpretable in terms of the measure and scale of the underlying data.

We used T-SNE analysis to map results from all lumbar levels to a two-dimensional space and therefore identify possible effects on overall lumbar loading. We qualitatively investigated data scattering for different model configurations and potential patterns due to applied loading and morphological factors. Concisely clustered data indicate similar loading overall lumbar levels, while high scattering data represent vastly differing loading. T-SNE analysis for different model configurations showed clear load case-specific clustering for combined loading and compression for each model configuration as well as in the full dataset, including all models (Figure 3). Although semi-individualized models form closely grouped clusters for each load case, transitions between clusters in highly individualized models are rather smooth, indicating that the load cases are less determining for resulting loads if models include more individual parameters. Anterior–posterior shear forces, primarily resulting from 30° flexion, tended to form a single cluster, while the resulting groups for unloaded and loaded upright standing merged into one another with a pronounced overlap for neutral and loaded standing with 10 kg in front of the chest. However, this effect was not present in models with a uniform spine, which showed concise clustering for respective load cases. Furthermore, the patterns in the results from those models indicated that the results are highly dependent on one intrinsic factor.

**FIGURE 4:**
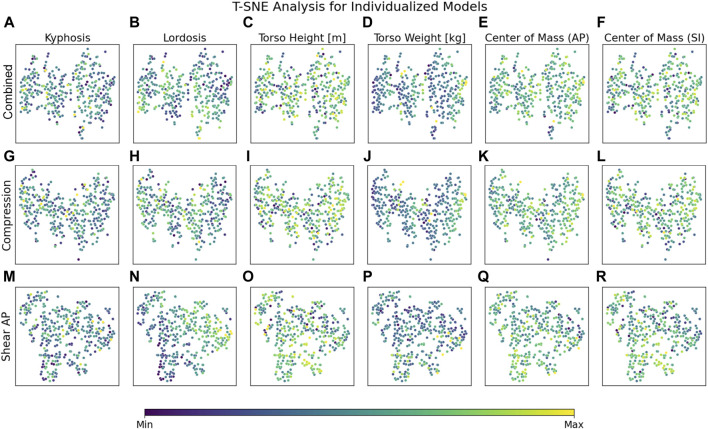
T-SNE plots for combined **(A–F)**, compression **(G–L)** and anterior-posterior shear **(M–R)** loading in highly individualized models. T-SNE plots based on individualized models show clear clustering for 30° flexion and for LL across all load cases based on anterior–posterior shear forces **(N)**. The effects of other parameters need to be investigated in detailed level- and load-case-specific analyses. *X* and *y* axes represent a virtual space that serves for projection and is not directly interpretable in terms of the measure and scale of the underlying data.

Regarding the considered morphological parameters, a strong gradient for LL in anterior–posterior shear forces overarching all load cases could be detected (Figure 4N). Less pronounced but still notable were the effects due to the anterior CoM location (Figure 4Q) and superior CoM location (Figure 4R). Detailed overarching effects of other parameters on lumbar loads could not be identified conclusively over all load cases (Figure 4) and needed to be investigated in detailed level- and load-case-specific analyses.

### 3.2 Multiple regression

We carried out multiple regression analyses for each load case and lumbar level to investigate the potential effects of various morphological parameters on lumbar loading. For the sake of clarity, we will focus on exemplary results at the L4/L5 level under upright standing load conditions, while briefly addressing other load cases. The results will be discussed specifically for compression forces and anterior–posterior shear forces while specifically emphasizing the changes due to model configuration. Note that a negative *β* in compression indicates unloading, while in anterior–posterior shear forces, it indicates anterior shifting of the load. Descriptive statistics on the absolute L4/L5 loading from the simulations are provided in Table 2.

**TABLE 2:** Statistics on simulation results, exemplary for level L4/L5.

Compression [N]									
Load case	Model configuration	Count	Mean	Std	Min	25th Perc.	50th Perc.	75th Perc.	Max
Neutral	Indiv	93	604.8	115.7	405.0	518.0	578.3	671.6	933.3
	uniTorso	92	584.0	63.8	479.1	539.0	572.6	622.1	799.2
	uniSpine	93	589.5	104.4	367.9	522.6	571.8	643.0	893.4
30° Flexion	Indiv	88	1,650.9	379.2	1,073.8	1,424.6	1,548.3	1764.3	3,625.2
	uniTorso	89	1,543.4	265.3	1,325.0	1,437.3	1,487.7	1549.3	3,494.7
	uniSpine	93	1,644.0	379.0	921.7	1,405.8	1,586.7	1789.6	2,867.2
10 kg, 25 cm	Indiv	93	964.0	152.5	692.3	850.8	928.6	1059.5	1,393.8
	uniTorso	93	961.1	79.6	816.2	914.8	949.9	989.8	1,291.7
	uniSpine	93	1,004.4	159.1	707.0	893.8	981.4	1090.9	1,516.0
10 kg, 55 cm	Indiv	93	1,677.1	268.7	1,241.2	1,507.7	1,639.8	1796.1	2,755.5
	uniTorso	93	1,655.4	166.9	1,381.4	1,550.3	1,622.7	1696.8	2,326.9
	uniSpine	93	1,738.6	341.4	1,211.0	1,501.6	1,649.7	1879.6	2,895.7
Shear AP [N]									
Neutral	Indiv	93	−99.6	84.9	−312.2	−146.6	−95.9	−42.5	119.4
	uniTorso	92	−92.0	79.1	−334.5	−137.9	−90.3	−35.9	89.6
	uniSpine	93	−125.6	16.1	−169.9	−135.3	−124.2	−116.2	−79.4
30° Flexion	Indiv	88	−108.0	167.8	−734.5	−169.0	−98.0	−3.6	264.1
	uniTorso	89	−101.1	155.3	−777.4	−156.5	−95.7	−5.3	247.0
	uniSpine	93	−99.5	51.3	−522.1	−108.1	−100.9	−89.6	62.8
10 kg, 25 cm	Indiv	93	−120.6	107.3	−476.1	−166.2	−105.6	−46.9	159.2
	uniTorso	93	−115.9	106.2	−522.0	−167.6	−106.2	−50.3	136.1
	uniSpine	93	−136.7	25.6	−262.8	−146.3	−131.3	−119.5	−89.5
10kg, 55 cm	Indiv	93	−219.8	179.0	−916.4	−294.9	−202.1	−95.2	275.2
	uniTorso	93	−213.6	183.7	−978.9	−294.6	−203.1	−93.0	240.4
	uniSpine	93	−212.4	48.0	−517.9	−232.0	−220.8	−198.6	−47.6

Count is the number of successful optimizations. Note that 93 is the maximum number of optimizations carried our per load case and model configuration.

#### 3.2.1 Effects of torso weight and distribution

**FIGURE 5:**
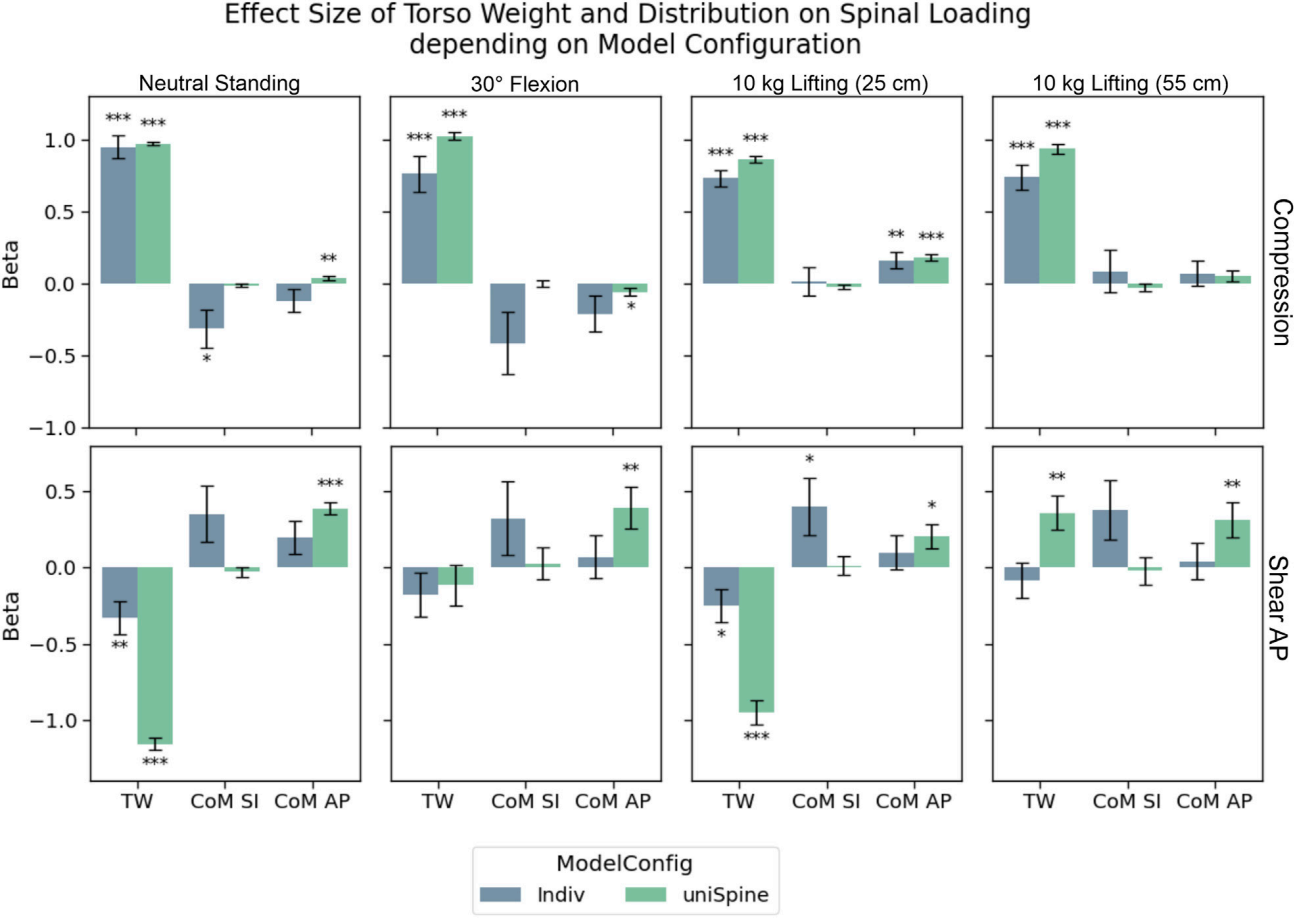
Effects of torso weight and distribution on compression and anterior–posterior shear in L4/L5. Significance levels were set to 0.05 (*), 0.01 (**), and 0.001 (***), and error bars depict the standard error of the estimate. Regarding anterior–posterior shear forces, including more individualized parameters in the models (Indiv) leads to a decrease in effect strength and even loss of significance, while effects are highly significant in semi-individualized models (uniSpine). Similar but less pronounced effects could be detected for compression.

During lifting tasks, TW showed highly significant strong effects (p < 0.001) on L4/L5 compression during for all model configurations (Figure 5). Effect strength tends to decrease with an increased degree of model individualization, especially in more demanding load cases such as flexion and lifting tasks. In comparison, the left of mass location barely influenced compression forces.

TW led to more pronounced anterior shear forces for neutral and moderately loaded upright standing (10 kg, 25 cm) for both model configurations, while only significant correlations could be detected between TW and posterior shear forces while lifting 10 kg with stretched arms. Significant effects of the calculated left of mass in the sagittal could only be detected for the anterior position in uniSpine models, increasing posterior shear forces. No such effects were observed for Indiv models.

#### 3.2.2 Effects of sagittal alignment and torso height

**FIGURE 6:**
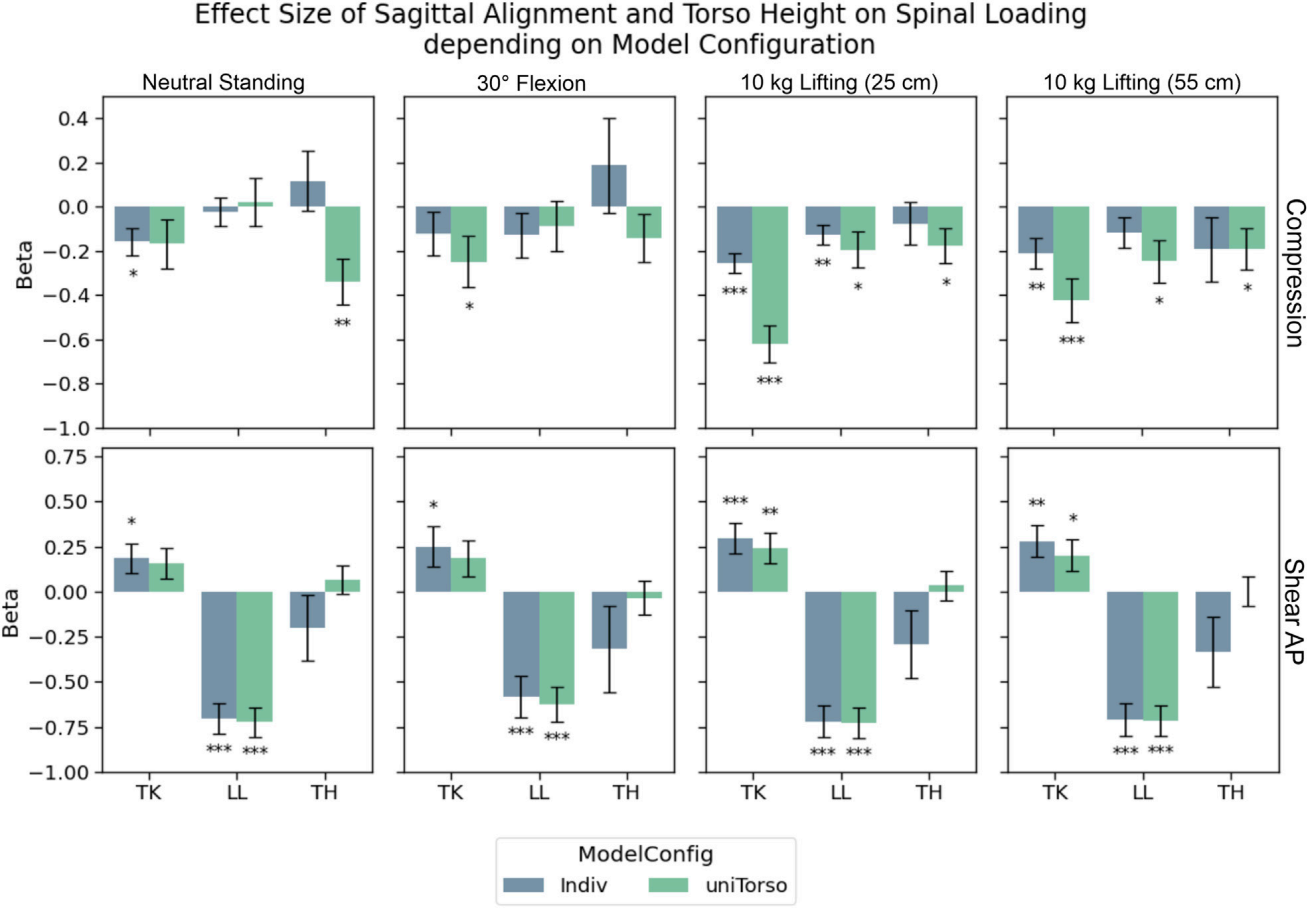
Effects of TK, LL, and TH on compression and anterior–posterior shear forces in L4/L5. Significance levels were set to 0.05 (*), 0.01 (**), and 0.001 (***), and error bars depict the standard error of the estimate. Effect strength for TK and LL decreased for compression forces, when models were highly individualized (Indiv) compared to semi-individualized (uniTorso).

For compression forces, strong unloading effects could be correlated to TK in both model configurations during loaded upright standing (Figure 6). However, effect strength decreased notably in Indiv models compared to uniTorso models. LL and TH showed significant weak effects for those models, while most of these effects were no longer detectable in Indiv models.

For anterior–posterior shear forces, LL showed the strongest effects for both model configurations. In this study, the respective effects hardly differ depending on the degree of individualization. Looking at the influence of TK on shear forces, significant correlations were detected in terms of a posterior shift of force for increased TK. These effects were even less pronounced in uniTorso models than in Indiv models. Similar trends could be observed for other load cases, whereas no more significant effects of TK could be observed in uniTorso models.

#### 3.2.3 Fit of the applied regression models

Based on the coefficient of determination *R* ^2^ we evaluated, we determined to what extent the applied multiple regression analyses were able to explain the observed variability (Table 3). For uniSpine models, multiple regression with only three independent variables (TW, CoM AP, and CoM SI) was able to explain the variability almost completely for all load cases (*R* ^2^ > 0.93). In comparison, for uniTorso models, the applied regression models showed a rather poor fit (*R* ^2^ < 0.47). For models, *R* ^2^ during 30° flexion was notably lower than in other load cases.

**TABLE 3:** Summary of *R* ^2^ values from regression analysis in L4/L5 during neutral standing (neutral), 30° flexion, lifting of 10 kg in front of the chest (10 kg, 25 cm), and with stretched arms (10 kg, 55 cm).

Model configuration	Independent variables	Load case	R^2^ (F_Compr_)	R^2^ (F_ShearAP_)
Indiv	TK, LL, TH, TW, CoM AP, CoM SI	Neutral standing	0.75	0.54
		30° flexion	0.44	0.29
		10 kg, 25 cm	0.87	0.52
		10 kg, 55 cm	0.70	0.48
uniTorso	TK, LL, TH	Neutral standing	0.11	0.48
		30° flexion	0.09	0.32
		10 kg, 25 cm	0.47	0.47
		10 kg, 55 cm	0.29	0.45
uniSpine	TW, CoM AP, CoM SI	Neutral standing	0.99	0.92
		30° flexion	0.96	0.11
		10 kg, 25 cm	0.97	0.69
		10 kg, 55 cm	0.93	0.38

## 4 Discussion

We investigated how model individualization affects results from musculoskeletal modeling studies on lumbar load estimation. One objective was to determine whether significant effects and clear trends can still be identified despite the increased variance in the results that come with increased model individualization. Furthermore, we aimed to investigate how the effect strengths of single parameters change compared to their respective results in semi-individualized models. Our study indicates that model individualization in combination with large patient cohorts holds the potential to obtain statistically significant results on relevant influencing factors on spinal loading while considering multiple aspects and their interactions. When comparing to semi-individualized models, we could detect changes in effect strength and significance for single influencing parameters, which might lead to misconceptions on the relevance of those parameters for spinal loading when only considering strongly simplified models.

### 4.1 Results from multivariate analysis

Load case-specific T-SNE analysis showed that for all degrees of individualization, similar trends could be detected (Figure 3). However, it is noticeable that the clusters from simulations with semi-individualized models (uniTorso and uniSpine) were considerably more compact than those from highly individualized models (Indiv). This already shows how different degrees of individualization in the model can influence the results. Considering only semi-individualized models could lead to the assumption that different load cases will result in clearly differing lumbar loading. However, including more individual parameters diffuses the cluster and thus weakens this apparent correlation while still preserving the initial trend.

For detailed analysis, we performed multiple regression analyses on simulation results for each load case and level separately. In this context, we focused on the potential effects of torso weight and distribution, as well as sagittal alignment and torso height. In agreement with the literature, TW had the strongest effect for both model configurations (Han et al., 2013; Hajihosseinali et al., 2015; Ghezelbash et al., 2016b). Compression forces were highly affected by TW for both the uniSpine and Indiv models, with decreasing effect strength when individualization was increased. Thus, although both model configurations indicate a relevant influence of TW, one might overestimate the effect of torso weight on lumbar loading when only considering generic models.

Regarding the effects of LL on anterior–posterior shear forces, the tilting of the lumbar vertebrae might most likely be one explanation for this observation. However, this strictly geometric explanation does not address the effect of TK on lumbar loading. Although the observed effects of LL (decreasing compression and increasing anterior shear) are in accordance with the literature (Müller et al., 2021), the observed effects of TK did not support findings from published studies, stating that spinal compression forces increased with increasing TK, with the most pronounced effects in the thoracolumbar and lumbar regions (Bruno et al., 2012). In our study, no significant correlation between TK and compression could be found for the T12/L1 level for both the Indiv and uniTorso models, while significant unloading effects were detected for L4/L5. Apart from the effect of posture, which was additionally stated by Bruno et al. (2012), one reason for this discrepancy could be that increased TK might also be correlated with other morphological factors, such as LL, and therefore leads to changes in muscle activity, which could not be assessed using generic models, where changes in TK are specifically induced without including other possible influences that might come along. To check for such a correlation, we performed linear regression analysis, resulting in a moderately significant correlation between LL and TK (*β* = 0.4) but a low *R* ^2^ (0.16), indicating that only a small part of the variability of thoracic TK can be correlated to LL. In addition, an influence of spinal alignment on the lever arms of the back muscles is conceivable, leading to potentially altered muscle activation and, therefore, changes in resulting lumbar loading. This, however, goes beyond the scope of this study and should be investigated separately.

Significant, slightly unloading effects of TH could mainly be observed for compression forces in upright standing in models with a uniform torso. This effect vastly disappeared in individualized models, making TH the least relevant parameter for lumbar loading in the present study. Assuming that taller subjects tend to have larger vertebrae and, therefore, larger lever arms of respective muscles, leading to decreased necessary muscle forces, could be one possible explanation for this slightly unloading effect (Han et al., 2013; Ghezelbash et al., 2016b). However, this correlation was not specifically investigated in this study and should be addressed in future work.

Overall, a decrease in effect strength and significant correlations with increasing model individualization could be detected, which supports the thesis that vast model simplification can lead to an overestimation of the influence of single parameters. Most striking is the observation that rather strong significant correlations were detected for the effect of TW in anterior–posterior shear forces, which either vanished completely or decreased strikingly with increased model individualization. Anterior–posterior shear forces, therefore, indicate that there are other strong influences that were not considered in this study. This observation underscores the relevance of considering multiple aspects to draw meaningful conclusions from numerical simulation studies.

**FIGURE 7:**
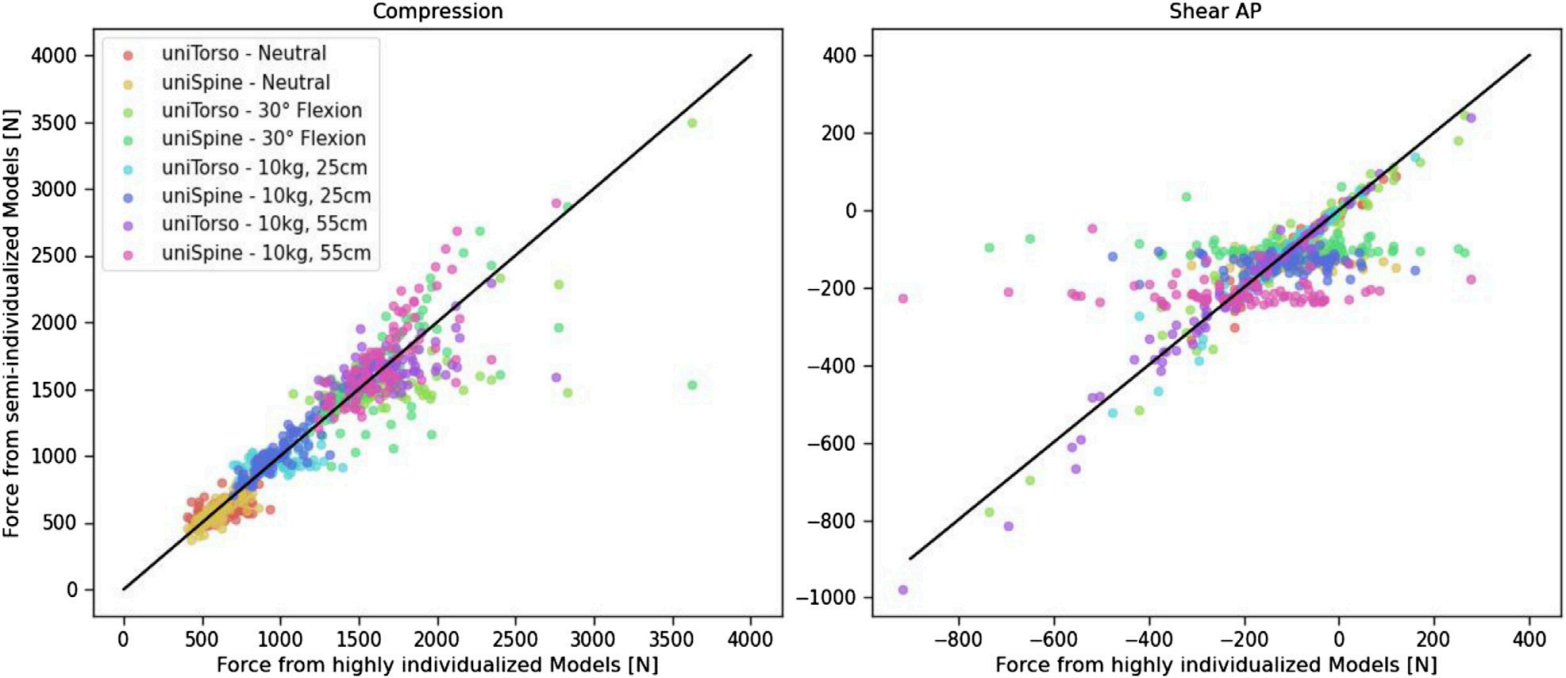
Comparison of generated forces from highly and semi-individualized models. The straight black line represents the perfect fit and is only added for orientation.

Comparing the generated forces from highly and semi-individualized models (Figure 7) showed that uniSpine models, in particular, led to high deviation when compared to Indiv models. This effect is more evident for anterior–posterior shear forces, which remain in the same range despite different body weights. This can be observed in different load cases. This emphasizes the relevance of including individualized spines in musculoskeletal modeling when it comes to analyzing large, diverse patient cohorts. Observed outliers could be assigned to individuals with morphological characteristics that differed strongly from the average. Thus, extreme compression forces were mainly observed in one subject with a calculated torso weight of more than 40 kg, which is an increase of almost 20 kg compared to the average of the cohort. In anterior–posterior shear forces, such extreme values were mainly detected in subjects with conspicuous features in terms of spinal alignment, such as scoliosis or hypolordosis.

### 4.2 Limitations and future perspectives

There are several limitations to this study. The used dataset with an overall sample size of 93 individuals and an average age of 70 years (Table 1) represents only a small sample of an elderly population and thus does not allow deriving conclusions for a general population. We did not directly include the influence of sex, age, or medical history in this study since respective information on their related effects on biomechanical properties was not available. However, associated parameters might be influential to spinal biomechanics in terms of individual mechanical properties of connective tissue, muscle morphology, or potential fat infiltration. Due to the lack of relevant information in our dataset, we included data from the literature (Panjabi et al., 1976; Christophy et al., 2012; Bayoglu et al., 2017). Therefore, referring to “model individualization” throughout this work, it should be emphasized that even highly individualized models here are only partly individualized, neglecting the variability in those parameters. Individual characteristics of the abdominal muscles, such as physiological cross-sectional areas or potential fat infiltration, can be associated with reduced capacity for muscle force production (Avesani et al., 2023) and, therefore, could lead to changes in spinal loading. In the literature, studies can be found that correlate these parameters to spinal degeneration and low back pain (Shi et al., 2022; Liao et al., 2024). Thus, they should be considered in individualized musculoskeletal modeling of the torso in the future. Although respective muscle-related parameters can be derived *in vivo* from imaging data (Niklasson et al., 2022), the mechanical properties of passive structures can currently only be determined with the help of *in vitro* studies (Panjabi et al., 1976; Pintar et al., 1992; Ashton-Miller and Schultz, 1997; Heuer et al., 2007; White, 2022), which require the isolation of the structure of interest to mount them in respective testing machines and therefore cannot represent the mechanical properties of the modeled subject. In order to obtain consistent datasets for biomechanical models, alternative, noninvasive methods must be developed to determine these parameters in large subject cohorts, e.g., using a combination of experimental studies, multimodal imaging, and artificial neural networks (ANNs), as suggested in earlier publications (Lerchl et al., 2023; Nispel et al., 2023). Furthermore, we neglected the effects of intraabdominal pressure (IAP) on spinal biomechanics. However, individual IAP measurements are usually carried out via the urinary bladder and require standardized conditions, which is why respective information is not accessible in large patient cohorts (Malbrain et al., 2006). However, the biomechanical relevance of the IAP has been subject to several studies in the past, stating its unloading and stabilizing effects on the spine (Arjmand and Shirazi-Adl, 2006; Mokhtarzadeh et al., 2012; Liu et al., 2019b; Guo et al., 2021). Thus, the IAP should be included in future studies, although not necessarily in a patient-specific manner.

Apart from general model-related limitations, which are discussed in detail by Lerchl et al. (2022), we want to emphasize one major limitation related to muscle modeling. Muscles are modeled as simple point-to-point actuators acting on a straight line between the origin and insertion of the respective fascicle. Considering especially multi-articulate muscles, this can induce bias, e.g., due to individual alignment or vertebral geometries. For example, in models with a pronounced LL, this can lead to lever arms that are considerably larger compared to models that redirect muscle fascicles along the spine. In the future, this should be addressed by including via-points along the spine to ensure realistic lines of action and, consequently, lever arms.

Complex models require the conscientious application and reflection of appropriate analyses. Evaluation of the coefficients of determination (*R* ^2^) showed that most of the variability in uniSpine models could be explained by TW and CoM, while *R* ^2^ decreased strikingly for uniTorso models. This can most likely be explained by the fact that the included individual representation of the trunk weight as simple point masses in a calculated left of mass does not induce further variability in the models apart from the parameters considered in the regression (TW, CoM AP, and CoM SI). However, individualization of the spine during this study included more parameters than only TK, LL, and TH, such as individual vertebral geometries. For one thing, this leads to a variation in the deformation of the included passive structures, namely, the intervertebral disc and the paraspinal ligaments, during joint deflection. The elastic properties produce a mechanical response in the form of forces and moments in the respective structures. In combination with individual vertebral geometries, this will result in individual additional loading for each subject and, therefore, individual loading of the intervertebral joints. On the other hand, individual vertebral geometries may affect lever arms for muscles and therefore muscle activation, ultimately resulting in lumbar loading, which could also be the reason for slightly unloading effects with increased TH. This might be one reason for the poor fits detected during 30° flexion for model configurations with individualized spines. It is noticeable that models that neglect the influence of TW (uniTorso) generally show the poorest fit of the regression model. The overall decrease in the *R* ^2^ value could be an indicator that TW is the most important determinant of spinal loading. If this is neglected, other parameters, such as vertebral geometries, become more evident and thus reduce the proportion of variability explained by the applied regression model. In the future, it will be essential to examine the underlying datasets in depth with regard to those parameters and potential interrelations between them prior to simulation in order to enable a profound and responsible interpretation of the intended correlation analyses.

Finally, an ultimate evaluation of the different approaches regarding their accurate representation of the *in vivo* biomechanics of a diverse population cannot be made directly. We validated our highly individualized model of a single male based on spinal loading and muscle activation of few subjects from *in vivo* data in a previously published study (Lerchl et al., 2022), but respective data are usually available only for small sample sizes due to the invasive character of *in vivo* measurements. Therefore, a population-based validation of the models in their different configurations is not feasible. However, there are several indications in the literature that model individualization in biomechanics leads to more accurate and realistic results (Akhundov et al., 2022; Davico et al., 2022; Meszaros-Beller et al., 2023). Thus, Davico et al. (2022) explored the influence of individualization in neuromusculoskeletal knee models of children. Based on experimentally derived data, models with different degrees of neuromusculoskeletal individualization were developed, and the obtained muscle and joint reaction forces were evaluated regarding physiological plausibility. In that context, the authors concluded that personalization of musculoskeletal anatomy and muscle activation patterns had the largest overall effect (Davico et al., 2022). Furthermore, the diversity of potential causes for spinal degeneration also supports the assumption that the strong reduction of individual influencing factors does not do justice to the complexity of spinal biomechanics (Kalichman et al., 2017; Kalichman et al., 2009). Thus, we are convinced that consideration of biological variability in musculoskeletal modeling is necessary and will increase a profound understanding of the complex interaction of various parameters influencing spinal loads and eventually leading to spinal degeneration due to overloading. One possible way to deal with such limited validation possibilities is to investigate potential correlations between spinal loading and clinical parameters, such as possible degenerative changes, based on large-scale, longitudinal studies (e.g., the German National Cohort). This can help us understand whether and how morphological and biomechanical characteristics can actually lead to mechanical overloading and, eventually, spinal degeneration.

## 5 Conclusion

In this study, we systematically investigated the effects of different degrees of individualization in multi-body models of the spine on generated lumbar loads and their potential correlations with morphological parameters. We used our validated pipeline for the automated generation of multi-body models of the trunk to create 279 models based on CT data from 93 patients. The influence on observed correlations was analyzed with respect to significance, effect strength, and explainability of observed variability in the results from static simulations based on multiple regression analysis. We were able to identify significant effects on lumbar loads for all load cases in models with different degrees of individualization. However, our results show that the degree of individualization influences the observed effect strength of individual parameters. For instance, in semi-individualized models, TW was the main influencing factor in both compression and shear loading. Including additional individualized parameters, however, showed that LL is more determinant for anterior–posterior shear forces and thus relatively reduces the importance of TW in this aspect. Based on the results of this study, we conclude that model individualization in combination with large patient cohorts holds the potential to obtain statistically significant results on relevant influencing factors on spinal loading while considering multiple aspects and their interactions. They can help identify potential risk factors or mechanical overloading based on data that represent the complexity of spinal biomechanics in a more realistic way than generic models can. However, a special focus should be put on the systematic and vastly holistic consideration of included parameters in applied analyses to be able to draw meaningful conclusions from studies including individualized models.

## Data Availability

The raw data supporting the conclusion of this article will be made available by the authors, without undue reservation.
